# Voltage-Controlled and Injector Layer Thickness-Dependent Tuning of Quantum Cascade Laser for Terahertz Spectroscopy

**DOI:** 10.1021/acsami.5c03040

**Published:** 2025-04-15

**Authors:** Mariusz Mączka, Grzegorz Hałdaś

**Affiliations:** Department of Electronics Fundamentals, Faculty of Electrical and Computer Engineering Rzeszow University of Technology, 35-959 Rzeszow, Poland

**Keywords:** terahertz wave imaging, quantum cascade laser, Green’s function methods

## Abstract

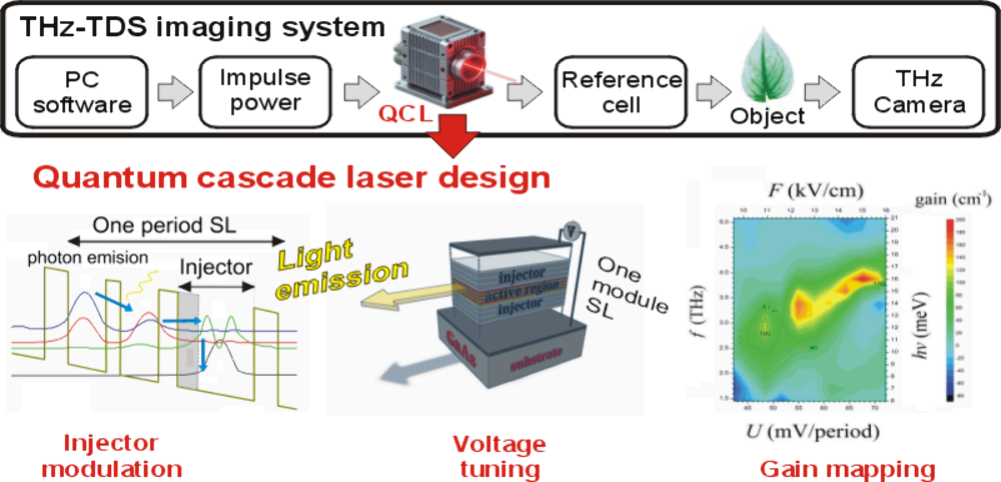

This work presents a numerical study of the tuning capabilities of quantum cascade lasers (QCLs) in terahertz imaging systems. This frequency range allows precise molecular identification without damaging the substance. QCL tuning is achieved by adjusting the power supply and the geometric dimensions of the injector region. Using the authors’ Infinite and Finite Model of Superlattice (IMSL and FMSL) approach, the model quickly predicts tuning trends, which are then validated with detailed radiation maps generated by the Real Space Model (RSM). Numerical results reveal a high sensitivity of the QCL optical gain to injector width variations, enabling the creation of either multiple separate tuning regions or a single continuous tuning region with minimal spectral shift.

## 1 Introduction

**Figure 1:**
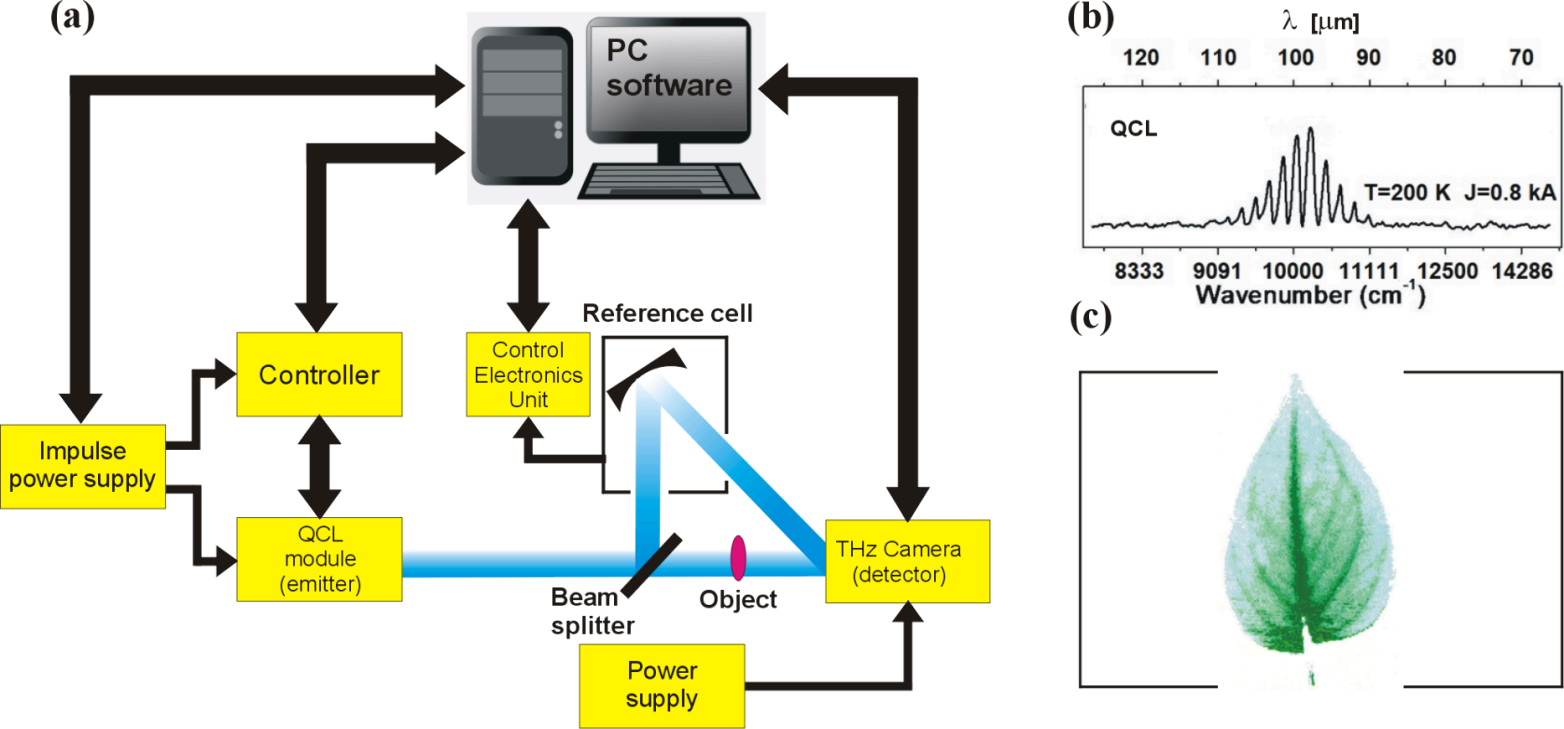
Illustration of an imaging method using a terahertz laser: (a) block diagram of a typical detection system with a QCL module as emitter; (b) energy spectrum of the emitted laser beam for typical power conditions; (c) image of the object obtained using a THz detector and camera.

Over the past few decades, terahertz radiation has emerged as a promising area of research, filling the gap between the microwave and infrared regions of the electromagnetic spectrum. Its unique properties, including its nonionizing nature and ability to penetrate various materials, make it an ideal candidate for a wide range of applications. One example of such an application is spectroscopy, which allows the analysis of the chemical composition of materials with exceptional sensitivity.^1,2^ Terahertz spectroscopy is also used in construction and architecture,^3−5^ environmental monitoring,^6−10^ pharmaceuticals,^11,12^ medicine,^13−15^ and even in the field of art conservation.^16−18^ The ability to precisely identify the molecular structure of a substance without causing any damage makes THz spectroscopes an indispensable part of airport security systems^19−21^ or the main tool for obtaining information about the internal structure of materials or objects.^22,23^ Such systems are based on the fact that forces arising from intra- and intermolecular interactions and their radiofrequency sensitivity to atomic structure.^24^ Intermolecular bonds cause rotation of the rigid body, movement of the center of mass and internal vibrational modes, which under the influence of THz radiation become the source of unique spectral features. These features allow the chemical identification of samples and the so-called chemical mapping in two^25^ or even three geometric dimensions^26^ in the time^27,28^ for frequency^29,30^ domain. An example of such a system is shown in Figure 1. The target object is raster scanned using the THz-TDS (Terahertz Time Domain Spectroscopy) technique.^31−35^ This technique uses short electromagnetic pulses of the order of picoseconds, allowing the measurement of signals with very high resolution and the observation of the dynamics of phenomena occurring in the tested materials over very short time intervals. The THz-TDS system consists of several key components that work together to provide precise analysis of the target. The main source of terahertz radiation is the QCL (Quantum Cascade Laser) whose beam is directed to the beam splitter. This element splits the beam into two parts: one part goes to the reference chamber, where the radiation parameters are analyzed under reference conditions, while the other part goes to the object under study and then to the THz camera, which acts as a detector. The object under investigation is located in the beam path between the beam splitter and the THz camera, which allows the analysis of its optical and structural properties. The reference chamber is connected to the control electrical units, which allow to control the measurement conditions and to verify the results against the reference standard signal.

The power supply and control system includes several key elements: Impulse Power Supply, which supplies power to the controller and the QCL module (terahertz laser emitter), Control Electric Units, which work with PC software to regulate measurement parameters and operating conditions of the reference chamber, PC software, which acts as a central control system and enables communication with the Impulse Power Supply, controller, Control Electric Units, and the THz camera. All system elements are interconnected in a way that allows precise control and data acquisition. The PC software acts as a master control module, enabling bidirectional communication with the Impulse Power Supply, Controller, Control Electric Units and THz Camera. Impulse Power Supply has a unidirectional connection with Controller and QCL Module, ensuring stable power supply to the system. In turn, the THz camera is powered by the power supply, which enables precise detection of the terahertz signal. The entire system allows precise imaging of the object under investigation, providing high quality data on its internal structure and optical properties.

**Figure 2:**
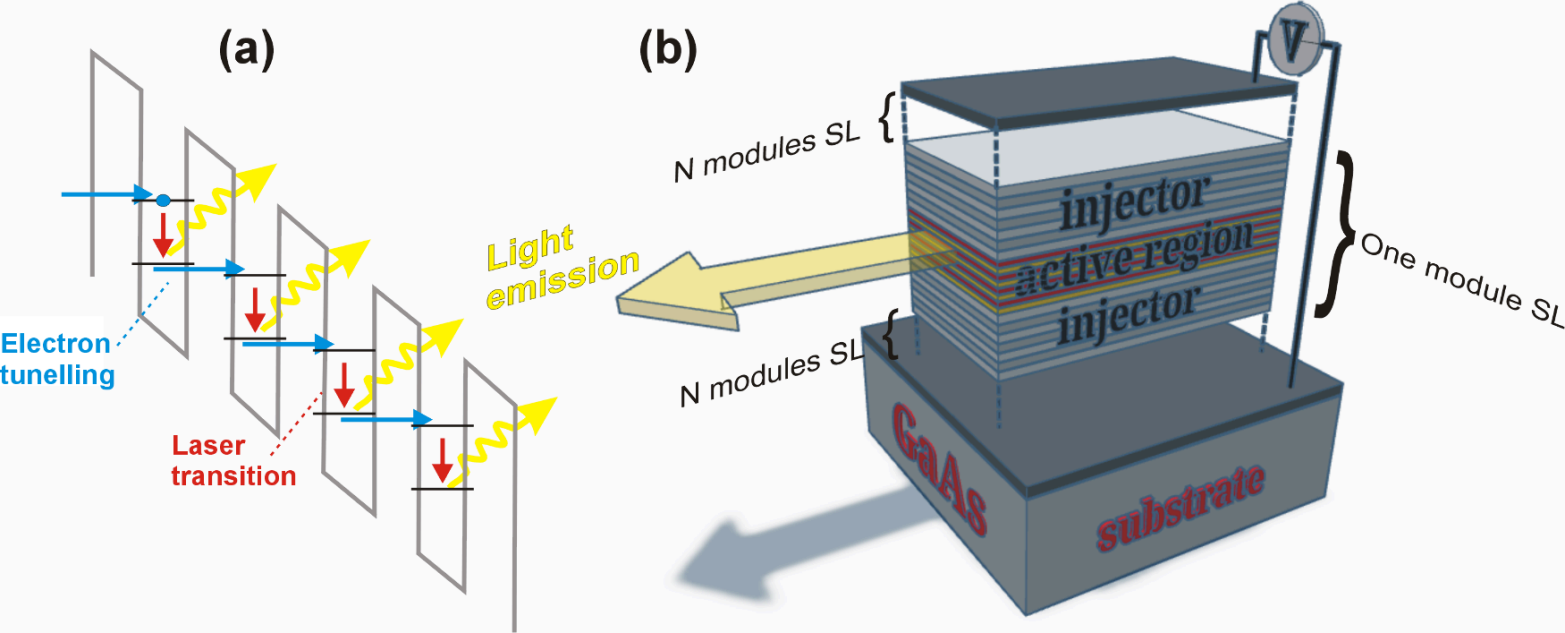
Idea of a quantum cascade laser: (a) one electron is the source of a cascade transition through the superlattice structure combined with multiple photon emission; (b) schematic structure of a laser consisting of many superlattice modules.

One of the main elements of the system described here is a quantum cascade laser, which has a relatively short but rich history.^36−38^ The schematic operation of this device is illustrated in Figure 2.

The device consists of many nanometer-sized alternating semiconductor layers with two different lattice constants (e.g., AlGaAs–GaAs), which form modules containing quantum wells (see Figure 2b). Such structures are called superlattices (SL) and are an essential part of QCL. The idea is that a one electron is the source of a cascade transition through the superlattice structure combined with the emission of many photons (see Figure 2a). Most importantly, it has become a leading emitter in the systems described here due to its relatively easy tunability and its ability to provide high spectral power densities at target frequencies above 2–4 THz, where many materials exhibit spectral absorption properties. As a result, research centers around the world are continuously working on improving and extending the capabilities of existing QCL structures,^39−41^ as well as finding new design solutions.^42,43^ A special role is played by work on increasing the operating temperature of lasers and their tuning ranges,^44−46^ which is crucial for improving their energy efficiency and enabling their operation at room conditions without the need for complicated cooling. In addition, the development of new semiconductor materials and advanced heterostructuring techniques allows the design of QCL structures with increased quantum efficiency, opening the way to their applications in spectroscopy, optical communication and biosecurity.^47−49^ An indispensable tool in these activities are numerical models and simulation programs,^50−53^ which significantly reduce the huge costs of developing and implementing innovative low-dimensional structures and also speed up the process of reaching the final design solutions of modern quantum devices.

Our research group has developed several complementary numerical models^54,55^ that can be used to simulate low-dimensional structures acting as emitters or detectors of electromagnetic radiation. The use of a specific model results from the nature of the research and the desire to optimize its duration. For example, the fast models IMSL (also known as Wannier Function Method - WFM)^56^ and FMSL^57^ are most often used for preliminary simulations for specific power supply conditions of the structure, in order to determine the range of computational parameters important from the point of view of the research. Then, for such a determined range, one can use the very versatile, but also time-consuming RSM.^58^

This paper presents numerical studies carried out to determine the tunability of the selected QCL structure described in,^59,60^ which could be used in a terahertz imaging system. The above structure was optimized by varying the thickness of the semiconductor layers responsible for extracting carriers from the active region of the laser and delivering them to the active region of the next device module (injector region). The calculations included checking the possibility of emitting THz radiation over a wide range of supply voltages, as well as determining the influence of temperature on the current–voltage characteristics of the device, taking into account all the necessary dissipation mechanisms.

## 2 Numerical Models of QCL

The simulation of quantum cascade lasers is a very complex process that requires a lot of computational resources and can take a very long time, depending on the models and objectives adopted. Therefore, the strategy adopted in this work is to use fast but less accurate models (IMSL, FMSL) to check the possibility of radiation emission in a wide range of supply voltage variations for different variants of injector layer thickness. On the other hand, the accurate RSM model has been used to determine accurate maps of laser radiation for selected, most interesting configurations of supply parameters and injector layer thickness.

### 2.1 IMSL and FMSL Models

To observe the location of the most important quantum states from the perspective of laser action, effective IMSL and optimized FMSL^61^ models were employed. The first one assumes infinite dimensions of the model and periodic boundary conditions. It uses the properties of Bloch and Wannier functions to create a basis of quantum states spanning three superlattice periods and relies on the nonequilibrium Green’s function formalism (NEGF) to determine the transport parameters of the structure. This approach involves solving the Dyson and Keldysh equations to obtain the delayed Green’s function, *G*^*R*^(*E*), and the Green’s correlation function, *G*^<^(*E*). The Dyson equation is written as
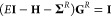
1where **H** represents the device Hamiltonian, **G**^*R*^ is the retarded Green’s function matrix, and **Σ**^*R*^ is the self-energy matrix. Initially, the self-energies, **Σ**^*R*^ were treated as constant diagonal elements iη, with the parameter η defined by
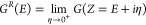
2

The Keldysh equation takes the following form:
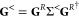
3where **G**^<^ is the correlation Green’s function matrix, and **Σ**^<^ denotes the self-energy matrix. In the initial state (thermodynamic equilibrium), this is a diagonal matrix with elements iη·*f*_*n*_ (*E*), where
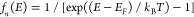
4where the *E*_*F*_ values are obtained using either the step function or a Büttiker probe-inspired^62,59^ approximation. In contrast, our approach introduces a novel method for calculating the *G*^*R*^ (*E*) and *G*^<^(*E*) functions, as well as the self-energies matrix, which, to the best of our knowledge, is unique among superlattice models. It is worth emphasizing that in the method the Hamiltonian device matrix is transformed from the energy and position representation to the pure energy representation. Thanks to this, its size is reduced by 2 to 4 orders, which significantly speeds up the calculations and makes it the fastest model we use. Therefore, a large number of simulations of structures with different configurations of supply voltages and injector layer thicknesses were performed on it.

The advantage of FMSL model is the use of polynomials to approximate the charge and potential distribution functions in a single layer of the superlattice. This allows for semianalytical solutions of the Schrödinger and Poisson equations, taking into account the changing potential in a single layer of the structure without the need to divide it into many subregions after applying a voltage to the system. Schrödinger equation in this approach has the form:
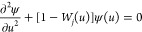
5where
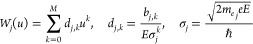
6

The dimensionless form of eq 5 was obtained after introducing dimensionless variables:
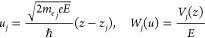
7where *V*_*j*_(*z*) is the potential function in the *j*-th layer of the superlattice and olutions are represented as a power series:
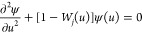
8

Poisson’s equation in the form
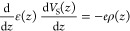
9where *ε* is dielectric function and *V*_*S*_(*z*) denotes the potential derived from impurities and unbalanced charge carriers, with the continuity conditions:
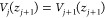
10
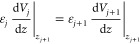
11is solved by assuming that the right-hand side of eq 9 is approximated by an *N*-degree polynomial in the *j*th layer of the superlattice in the form
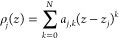
12

The approximation procedure consists in comparing the charge density function calculated in the previous iteration step with the function described by formula 12 at *N* + 1 selected points (nodes) of each structural layer. This leads to algebraic equation systems of *N* + 1 degree, the solution of which gives the values of the coefficients *a*_*j,k*_ A detailed description of the method mentioned here, together with sample calculation results, is described in the works.^56,57^

This model has been used to simulate the most interesting cases of input parameter configuration (supply voltage and injector thickness), selected after analyzing the results obtained by the IMSL method. The FMSL model allows taking into account the mutual interaction of a larger number of superlattice modules (IMSL considered 3 periods), which allows a better approximation of the structure consisting of several tens of modules.

**Figure 3:**
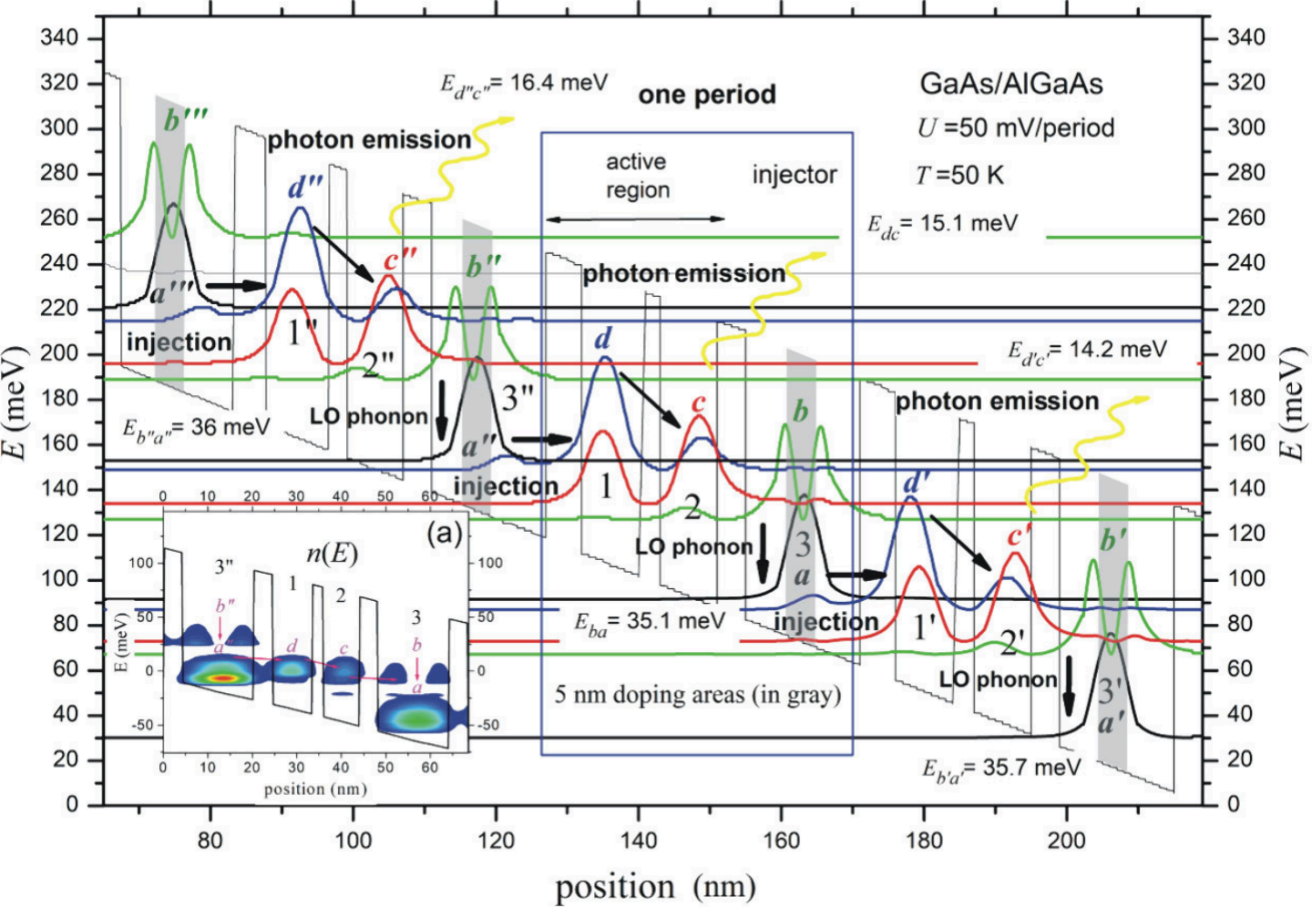
Basic electron transitions within four modules of the investigated QCL (#0) and the electron concentration for a single period of structure calculated at a voltage *U* = 50 mV/period and at temperature *T* = 50 K calculated by use of IMSL and FMSL models.

Example simulations performed using the IMSL and FMSL models are shown in Figure 3, which shows the test result of the QCL structure initially calculated for three superlattice periods (IMSL) and extended to four superlattice modules. These modules are marked as #0 in the above paper. The quantum states visible here were obtained for a temperature of *T* = 50 K and a voltage of *U* = 50 mV per period using the Modified Bisection Method (MBM) with a parameter of *N*_*L*_ = 50. Such a value of the *N*_*L*_ parameter is responsible for the number of analyzed compartments in the unit energy range, which provides high accuracy in the determination of eigenstates with optimal computational time. The algorithm of the method, together with a detailed description of its other parameters, has been published in the ref (61).

The choice of temperature was dictated by the well-known fact that for 50 K all dopants are ionized. In this case, it was possible to detect 60 eigenstates grouped in the range of 12 minibands. The most important eigenstates for the operation of the laser were then plotted. The position of the states in the energy domain was calculated with the accuracy defined by two parameters of the Transfer Matrix (TM) procedure, in which the wave functions are represented by power series. The first of these parameters (*n*_*KS*_ = 30) determines the number of terms in the power series within the procedure for calculating the eigenenergies, while the second (*n*_*KD*_ = 100) refers to the number of terms in the power series within the procedure for calculating the waveforms of the wave functions. A detailed description of the method and its parameters has been published in.^57^ Furthermore, the influence of the charge originating from the 5 nm doped areas located in the injector cavity (marked in gray) was taken into account, which was determined by the self-consistent solution of the Schrödinger and Poisson equations. Simulations in this regard were carried out according to the algorithm described in the paper.^57^

In the active region of the laser, which consists of two narrow (8.9 and 8.15 nm) quantum wells (numbers 1 and 2), two different states are distinguished: a high (*d*) state and a medium (*c*) state. These states, which are powered by electrons from the phonon well injector (3”) of the previous period, serve as the source of the photon transition *d*→c with energy *hν* = *E*_*dc*_. The electrons are then transported by the electric field to the extractor region (3), which also serves as an injector for the *d′* and *c′* states in the subsequent period of the structure. In the area of the phonon well (3), for the given supply voltage, there is a *b* → *a* a transition with energy *E*_*ba*_ = 35.1 meV. It is noteworthy that the energy of the *b′* → *a′* phonon transition is *E*_*b′a′*_ = 35.7 meV, while for the *b″* → *a″* transition it is *E*_*b″a″*_ = 36 meV. Consequently, we are dealing with an energy spectrum of phonon transitions analogous to the case of photon transitions. In the latter, the photon energy *d* → *c* has an energy of *E*_*dc*_ = 15.1 meV. However, for the *d′* → *c′* transition, we have *E*_*d′c′*_ = 14.2 meV, while the transition *d″* → *c″* gives the energy *E*_*d″c″*_ = 16.4 meV. It can thus be demonstrated that *hν*_*max*_, which corresponds to the energy of the transition *b″* → *a″*, is distinguishable from *hν*_*min*_, which represents the energy of the transition *d′* → *c′*. The distinction between these values provides insight into the width of the emitted energy spectrum, as illustrated in Figure 1b. This width, which is referred to in the work as Δ*ν̂* (cm^–1^), is commonly understood as the wavenumber. The calculated value of the wavenumber using the FMSL for four modules of the tested structure supplied with voltage *U* = 50 mV/period is Δ*ν̂* = 1610 cm^–1^. In contrast, the same parameter calculated for a structure containing ten modules increases to Δ*ν̂* = 7017 cm^–1^ and remains relatively constant with further increases in the number of QCL modules. The data obtained in this manner, validated by more precise calculations using the RSM, constituted one of the input parameters for subsequent design calculations, which will be described in the subsequent chapter.

Laser action is contingent upon population inversion, wherein the electron density in the *d* miniband exceeds their concentration in the *c* miniband. This phenomenon can be observed in the energy map of electron concentration shown in the lower left corner of Figure 2, marked as (a), which was prepared for the structure #0 supplied with a voltage of *U* = 50 mV/period at *T* = 50 K. The results demonstrate a markedly higher electron density around the *d* state relative to the *c* state, which ensures *d* → *c* photon transitions. Furthermore, the high electron density observed in the 3″ quantum well, resulting from the 5 nm doping layer at *N*_*d*_ = 6 × 10^22^ m^–3^, ensures that the energy levels of the *d* miniband are occupied by electrons injected by the electric field from the *a* miniband. Tunnelling processes occur between the injector well 3 and the well of the active region 2, which supply electrons to miniband *b*. These electrons are then transferred to miniband *a* through phonon transitions, which subsequently occupy the states. This miniband supplies the *d*′ state in the subsequent module. The sequence of charge carrier transport combined with multiple photon emission by the same electrons is therefore possible, and its quantitative value can be accurately calculated using the RSM, which will be described in the following chapters of the paper.

### 2.2 RSM Model

RSM is a semi-infinite model in which the multiquantum well alignment of the polarized superlattice structure is continued in the derivatives. It is assumed that the conduction band edge does not change outside the analyzed part of the structure. In this approach, the Keldysh and Dyson transport equations are solved assuming that the retarded Green’s functions can be written as
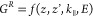
13where z and z′ are the real space coordinates. This is the main difference of this model from IMSL, as it does not require initial calculations of allowed minibands in the simulated structure. In contrast to IMSL, the quantum states in the superlattice can be obtained directly using the NEGF formalism. But on the other hand, the Hamiltonian matrices describing the device reach enormous sizes, which greatly increases the time of solving the transport equations and requires very large memory resources. Detailed differences between both models are described in.^54^

**Figure 4:**
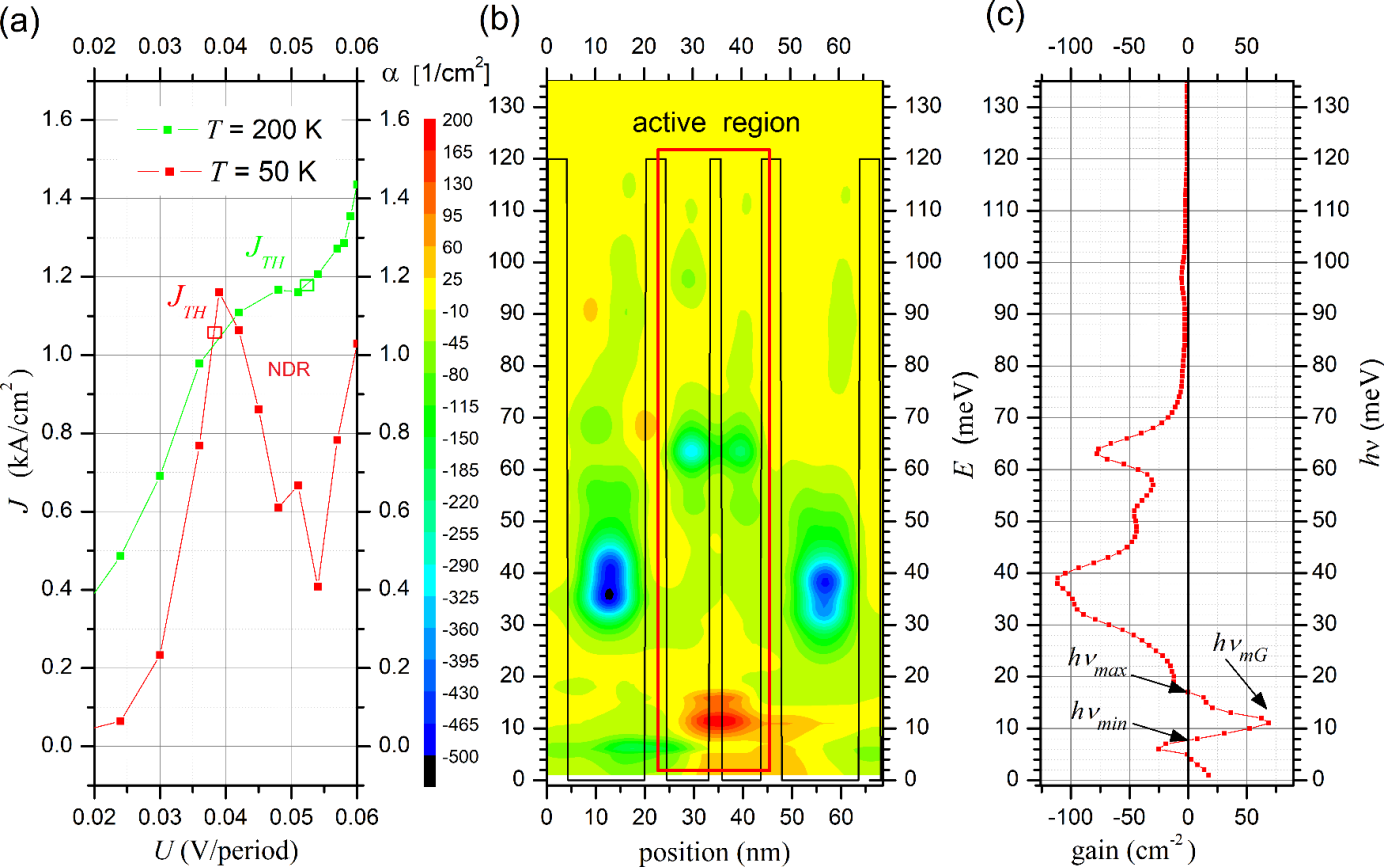
Simulation results of the QCL with RSM for the data in Table 1 for structure #0: (a) *I*–*V* characteristics with laser threshold current for *T* = 200 K and *T* = 50 K; (b) optical gain (α (cm^2^)) spatial-energy map; (c) dependence of sumed optical gain (cm^–1^) on the energy *hν* (meV) for voltage *U* = 45 mV/period.

Example QCL simulations using RSM are presented in Figure 4, were obtained using the parameters given in Table 1. The calculations take into account electron scattering due to the crystal lattice disorder (AD), interface roughness (IR), scattering on impurity ions (ID), acoustic and optical phonons (AP and OP, respectively) and electron–electron (E–E) interactions according to their implementation method, as described in the works.^58,63,64^ The *I*–*V* characteristics of the tested QCL are presented in Figure 4 a, where the dependencies of the current density *J* (kA/cm^–2^) on the supply voltage *U* (V/period) are plotted. These were calculated for temperatures *T* = 200 K and *T* = 50 K, respectively, and are plotted in green and red. The first of the aforementioned characteristics was used to compare the simulation with real measurements made in the work.^59^

**Table 1:** Basic Parameters of the QCL Simulation for the Results Presented in Figures 3–6

GaAs/Al_045_Ga_0,55_As QCL	well		barrier
effective mass, m*	0.067		0.104
bandgap, *E*_*g*_ (eV)	0.84		1.84
relative permittivity, *ε*_*r*_	12.85		13.8
structure layers, (nm) (bariers in bold)		#0: **4.3**, 8.9, **2.46**, 8.15, **4.1**, 16.0	
		#1: **4.3**, 8.9, **2.46**, 8.15, **4.1**, 15.4	
		#2: **4.3**, 8.9, **2.46**, 8.15, **4.1**, 16.6	
band offset (eV), Δ*E*_*C*_		0.12	
*n*_*dop*_ (cm^–3^)		6 × 10^16^	
LO-phonon energy (eV)		0.036	
deformation potential (eV)		5.89	
screening length (nm), *l*_*Debye*_		32	
no. of periods QCL		236	
temperature (K)		50, 200	

After calculating the point values for the given structure power conditions, it was found that the obtained simulation and measurement results converged, confirming a good fit of the model input parameters. The threshold value of current density *J*_*TH*_ ≅ 1.195 kA/cm^2^ was determined by taking into account losses in the waveguide as described in the aforementioned work, where *g*_sb_ ≅ 35 cm^–1^ was assumed as the threshold value. In the graph for *T* = 50 K, where the threshold current is *J*_*TH*_ ≅ 1.025 kA/cm^2^, the portion of the characteristic exhibiting negative dynamic resistance (NDR) is noteworthy, which is not observed during measurements (see work in ref (59)). The physical reasons for such a phenomenon resulting from the assumption of perfectly repeatable geometric dimensions of each superlattice module for the simulation have been described in detail in ref (65). Nevertheless, this does not significantly impact the subject of our research.

The map of the laser optical gain (α (cm^–2^)) visible in Figure 4b, calculated for a voltage of *U* = 45 mV/period and a temperature of *T* = 50 K, plotted against the background of the potential of the structure, reveals several crucial aspects of laser operation. (i) It can be employed to present electronic transitions between quantum states in both the energy and position domains in a quantitative manner. (ii) It illustrates the areas of radiation emission (positive value of the α parameter) and its absorption (negative value parameter α). (iii) It provides information indicating that photon transitions are essentially limited to the active area of the laser and that their intensity is the highest in the energy range corresponding to the position of the *d* and *c* states.

The values of the α parameter with respect to the position can be summed to plot the characteristics shown in Figure 4c. This figure illustrates that the values of energy *hν* for positive optical gain are in the range *hν*_*max*_ – *hν*_*min*_ = 10 meV, which gives Δ*ν̂* = 8065 cm^–1^. This approximately corresponds to the calculations obtained using FMSL, but in this case, it was achieved with much less computational time and computer memory. However, the precise results visible in Figure 4c allow the determination of the optical gain peak, which in this case occurs for *hν*_*mG*_ = 11 meV and has a value of *g*_*max*_ = 74 cm ^–1^. Based on the *hν*_*mG*_, trend lines for the change in laser radiation caused by changes in the thickness of the injector and the power supply conditions of the structure were determined and will be described in the following chapters of the work.

## 3 QCL Tuning

**Figure 5:**
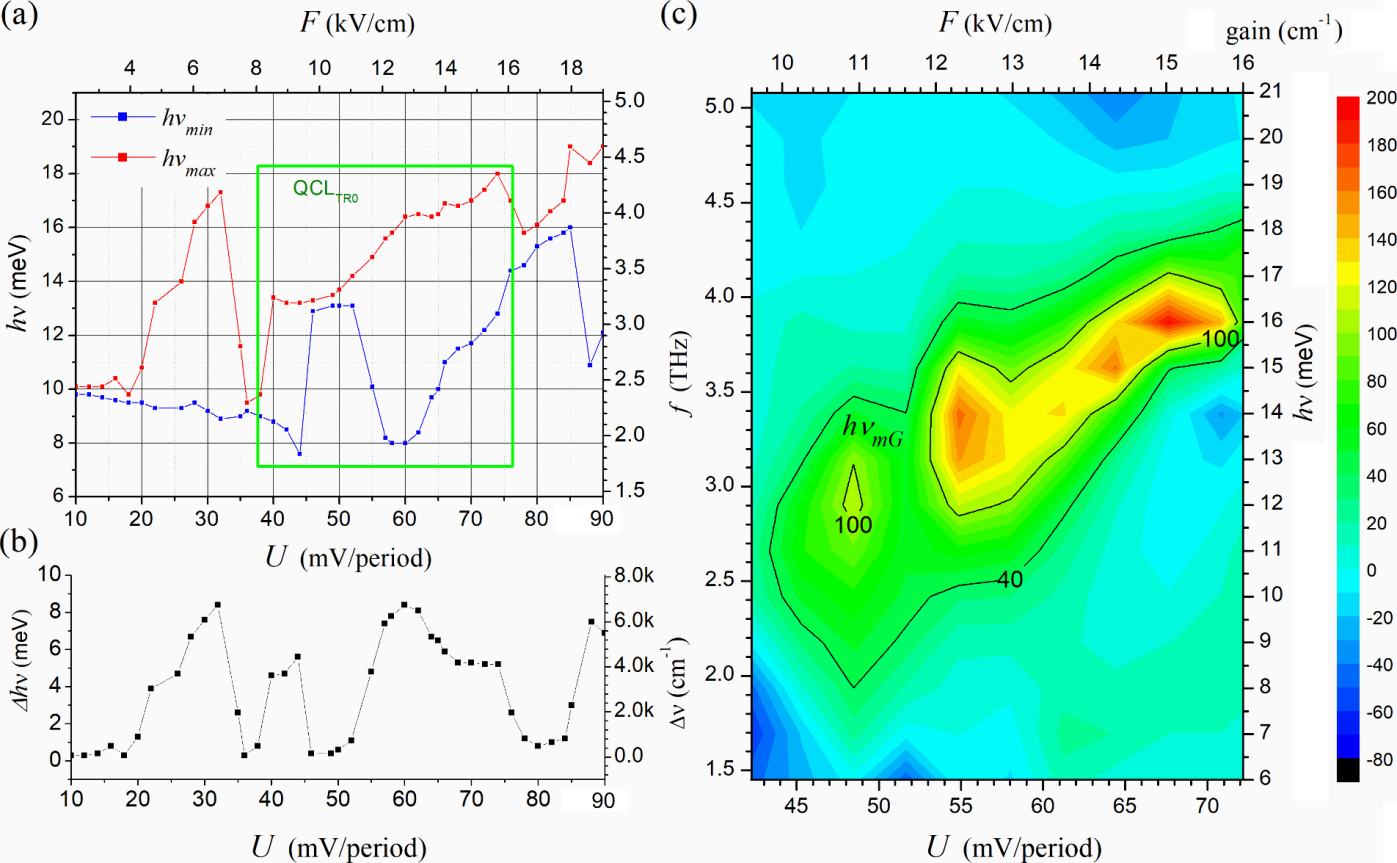
QCL tuning simulations for structure #0: (a) the minimum *hν*_*min*_ (in blue) and maximum *hν*_*max*_ (in red) values of the transition energies *hν* (meV), for the two observed minibands (d and c) and the width (Δ *hν*) of the radiation spectrum; (b) depending on the bias voltage *U* (mV/period) with the effective tunable area QCLTR#0F; (c) optical gain energy map plotted in the region covered around QCLTR#0F with the *hν*_*mG*_ trend line marked.

As demonstrated in the work,^59^ the range of waves emitted by terahertz lasers can be modified by adjusting the power supply conditions in an appropriate manner. Based on the aforementioned experiments, the operation of the selected structure was simulated in a wide range of supply voltages to assess its tunability in terms of use in THz-TDS imaging systems. The results of these simulations are presented in Figure 5, which were carried out for *T* = 50 K. Figure 5 a illustrates the borders (in the energy domain) of the highest (*hν*_*max*_) and lowest (*hν*_*min*_) values of photon transitions depending on the structure’s supply voltage.

The calculations were conducted using FMSL with the input parameters specified in Section 2 for ten superlattice modules spanning the supply voltage range from 0 to 80 mV/period. Two additional scales, *f* (THz) and *F* (kV/cm), have been introduced in the chart to facilitate the conversion of the results obtained for Δ*hν* (meV) and *U* (mV/period), respectively. The supply voltage range responsible for tuning the laser above the *J*_*TH*_ threshold has been specified, which is marked as QCL_TR#0F_ (Tunning Range #0 FMSL). The graph illustrates that in the QCL_TR#0F_ range, there are significant changes in the width of the radiation spectrum.

These changes are illustrated in part (b), where the dependence of Δ*hν* (meV) on the supply voltage *U* (mV/period) is plotted. These changes were converted into wavenumber values that can be read using an additional axis Δ*ν̂* (cm^–1^). As illustrated in the graph, the width of the radiation spectrum of the tested laser reaches Δ*ν̂*_max__#0F_ = 6800 cm^–1^ (see for *U* = 60 mV/period), which corresponds to wavelength changes in the range from *λ*_*min*_ = 77.5 μm to *λ*_*max*_ = 154.9 μm (Δ*λ* = 77.4 μm). As the supply voltage is increased beyond a value of *U* = 60 mV/period, the radiation spectrum narrows. However, the narrowest spectrum is observed in the range of *U* = 46–52 mV/period. The width of the spectrum drops even to Δ*ν̂*_*min*__#0_ = 323 cm^–1^ (for *U* = 49 mV/period), which corresponds to changes in wavelength in the range from *λ*_*min*_ = 91.8 to *λ*_*max*_ = 94.6 μm (Δ*λ* = 2.8 μm). The entire QCL_TR#0F_ range allows the laser to be tuned to a value of *f*_TR#0F_ = 1.9–4.4 THz, which corresponds to waves in the range λ_TR#0F_ = 68.1–157.8 μm.

A comprehensive illustration of radiation in specific energy ranges is shown in Figure 5c, which shows the gain map *gain* (cm^–1^) with respect to the device supply voltage *U* (mV/period). Calculations were performed using RSM, taking into account all major scattering mechanisms (AD, IR, ID, AP, OP, and EE) and assuming an energy resolution of 1 meV and the parameters given in Table 1. The chart shows that the highest radiation (gain > 100 cm^–1^) occurs for voltages *U* = 54–71 mV/period, where the device can be tuned in the range *f*_TR#0R_ = 2.9–4 THz, which corresponds to the wavelength λ_TR#0R_ = 74.9–103.3 μm. The maximum width of the radiation spectrum is observed near around the voltage *U* = 55 mV/period, where Δ*ν̂*_*max*_100*g*__ = 995.4 cm^–1^ (for gain > 100 cm^–1^) and Δ*ν̂*_*max*_40*g*__ = 5503.4 cm^–1^ (for gain > 40 cm^–1^). The results obtained are consistent, with a small discrepancy, with those predicted by FMSL, as the calculations do not take into account the losses occurring in the device’s waveguide and all scattering mechanisms. Consequently, it can be concluded that the FMSL is a useful tool for rapidly estimating the trend and tuning range of the QCL, which is then corroborated by the trend line (shown in black) visible in map (c) and designated as *hν*_*mG*__,_ relating to the optical gain maxima (see Figure 4c). The collapse observed around *U* = 55 mV/period is directly related to the narrowing of the spectrum, as illustrated in Figure 4a.

## 4 Modeling of QCL Injector Region

**Figure 6:**
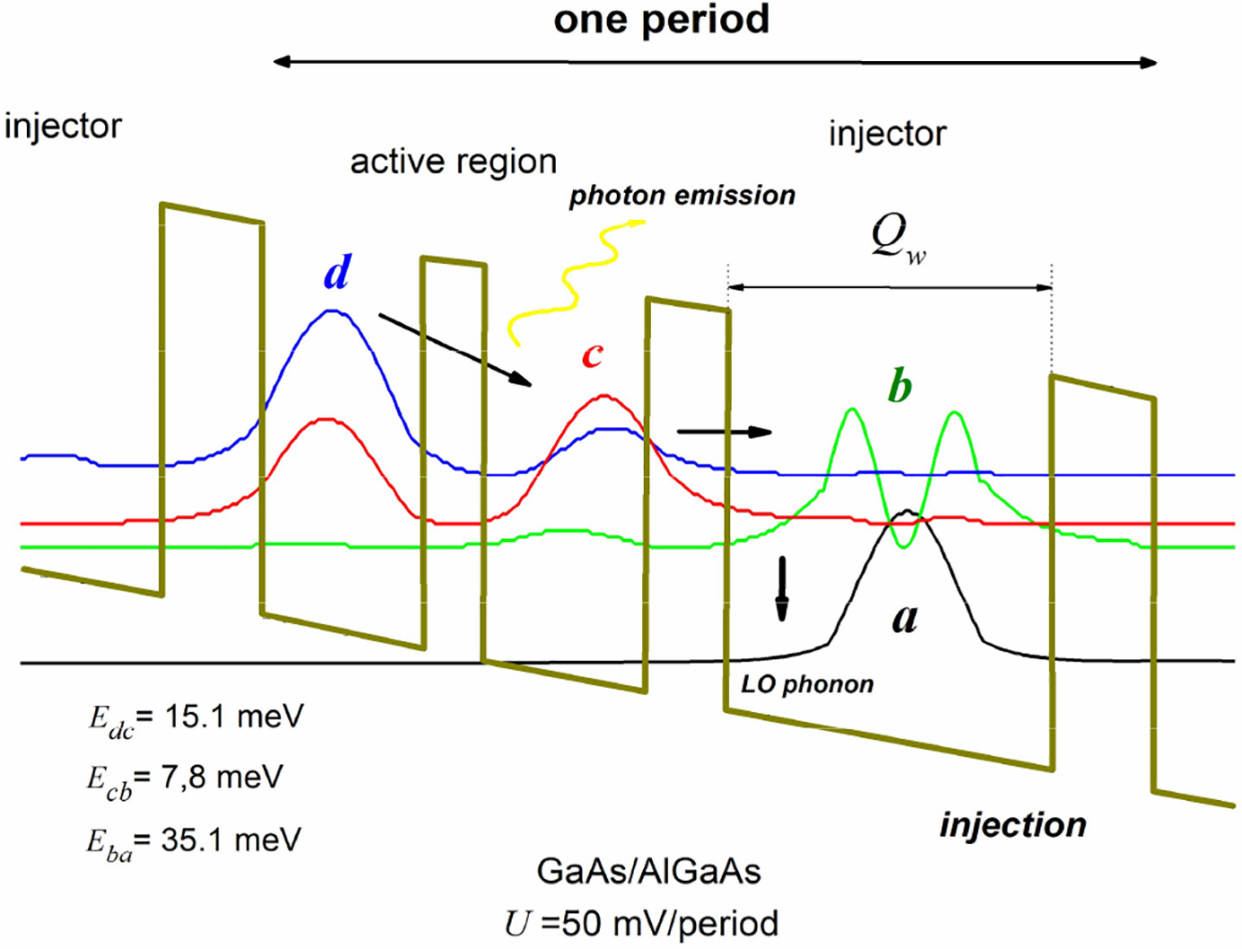
Modeling diagram of the QCL injector area with the considered states marked.

In their work,^59^ the authors report that the thickness of the injection and extraction barriers was optimized prior to fabrication of the device based on experiments conducted using simple Monte Carlo (MC) and Matrix Density (MD) models. From our perspective, the critical location of state b (see Figure 6), which is connected to states *d* and *c* by a small but visible coupling, may act as a bottleneck between the active area and the injector.

Therefore, it seems logical to investigate its impact on the tunability of the structure. Consequently, a study was conducted within the framework of the present article to evaluate the impact of *b*-miniband changes caused by varying the width of the injector cavity *Q*_*w*_ on the character of the radiation spectrum of the laser structure.

**Figure 7:**
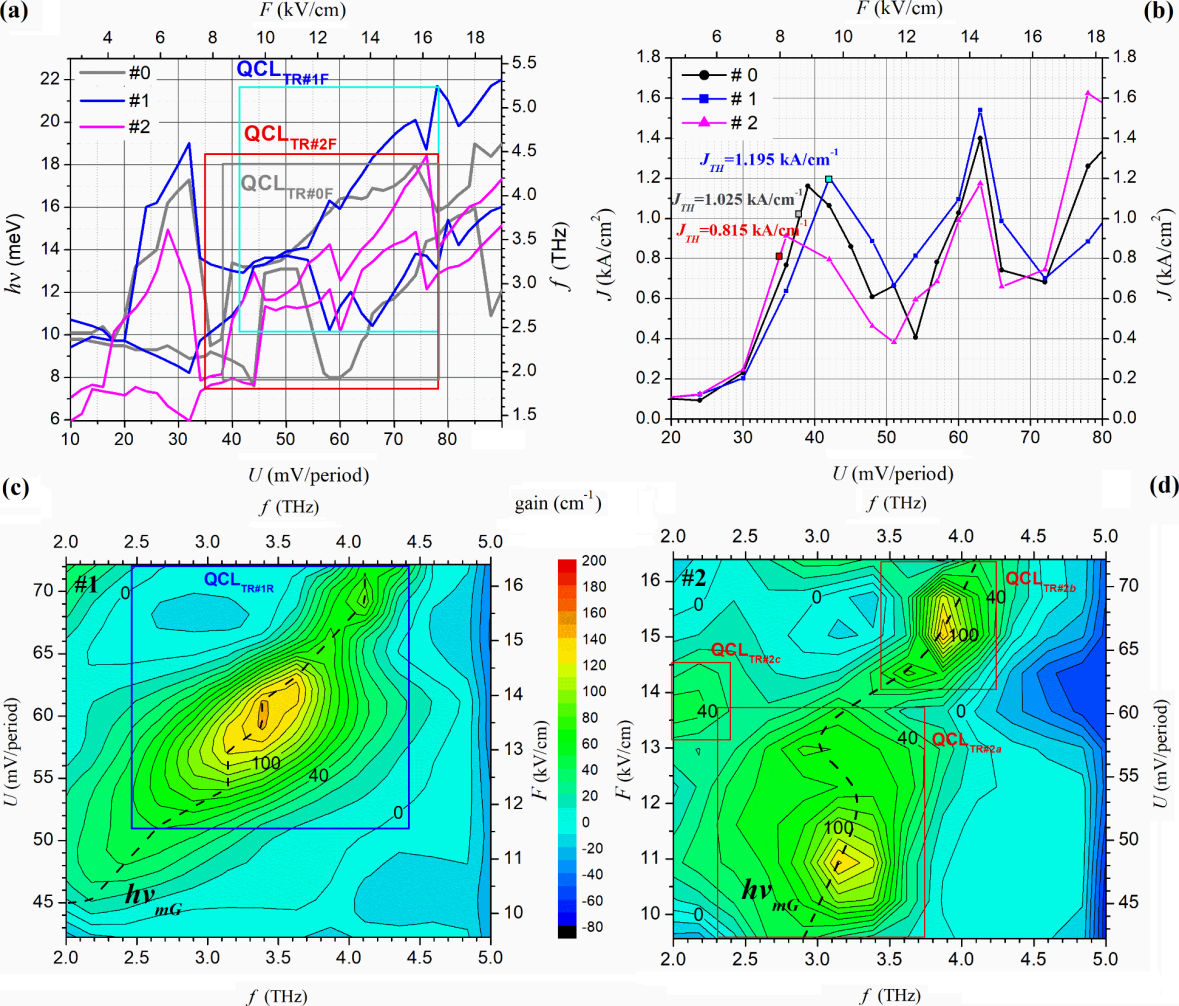
Fragments of current–voltage characteristics of the QCL with marked threshold currents for different widths of wells *Q*_*w*_ calculated using the RSM with the parameters listed in Table 1. Results (a) for increasing and (b) for decreasing the width of the well *Q*_*w*_. (c and d) Optical gain vs bias voltage and frequency maps for increased (#1) and decreased (#2) *Q*_*w*_ by one monolayer, respectively

The methodology of the research entailed the execution of expeditious and simplified simulations of the structure for reduced and increased widths of *Q*_*w*_ (±10%) in comparison to the original layer thickness (structure #0). Then, the most interesting cases were selected for detailed analysis, with precise calculations to include the most significant electron scattering mechanisms (AD, IR, ID, AP, OP, E-E). The two most interesting structures were created by changing the *Q*_*w*_ width by approximately two monolayer (±0.6 nm). These structures are designated as #1 and #2 in the work, and the most significant results presented in Figure 7 pertain to these structures.

Figure 7 a depicts the boundaries of the radiation spectrum, expressed by the energy of the emitted photons *hν* (meV), as a function of the supply voltage *U* (mV/period), for the narrowed (in blue) and widened (in red) wells of the injector. The boundaries are represented by the values of *hν*_*min*_ and *hν*_*max*_, which have been converted to their corresponding frequencies *f* (THz). Furthermore, the supply voltage has been converted to values of electric field and presented on an additional axis, *F* (kV/cm). The results were compared with those obtained when the injector well was represented by the original size (#0 in gray). The simulations in this section were conducted using FMSL with input parameters consistent with those described in Section 2, including a doping charge of *n*_*dop*_ = 6 × 10^16^ cm^–3^ and a temperature of *T* = 50 K. The graphs illustrate the tuning areas (QCL_TR#0F_, QCL_TR#1F_ and QCL_TR#2F_), which were defined based on the *I*–*V* characteristics observed in Figure 6b.

The threshold currents and corresponding supply voltages were observed to be limited from below the range of supply voltages that can be used to tune the laser. As illustrated, narrowing the injector well by one monolayer increased the laser threshold current to *J*_*TH*_ = 1.195 kA/cm^2^, while widening it in the same dimension lowered the threshold current to *J*_*TH*_ = 0.815 kA/cm^2^. Neither of these changes resulted in any visible alterations to the nature of the relationship, as evidenced by the persistence of two distinct areas of negative differential resistance (NDR) in both cases. It should be noted that the aforementioned current values were determined under the assumption of a temperature of *T* = 50 K and losses in the instrument’s waveguide at a level of *g*_sb_ ≅ 36 cm^–1^.

Narrowing the injector well by one monolayer (*Q*_*w*_ – 0.6 nm) resulted in a slight shift of the radiation spectrum in the higher frequency range and its broadening particularly for the high bias voltages (for *U* = 65–76 mV/period). This can be observed, for example, in Figure 6c or the voltage *U* = 70 mV/period, where these changes extend in the range *f*_*min#*__1F_ = 2.90 THz to *f*_*max#*__1F_ = 4.7 THz, which gives the width of the radiation spectrum Δ*f*_*#*__1F_ = 1.8 THz. It is maximum width of the radiation spectrum in this tuning area, converted to wavenumber this gives Δ*ν̂*_*max*__#1F_ = 6000 cm^–1^. For comparison, Δ*ν̂*_0F_ = 4275 cm^–1^ (see the gray graph for the same voltage) when converted gives us Δ*f*_*#*__0F_ = 1.28 THz, which corresponds to frequency changes *f*_*min#*__0F_ = 2.83 THz to *f*_*max#*__0F_ = 4.11 THz. In the remaining area of the QCL_TR#1F_ (for *U* = 43 – 65 mV/period) we observe a band cut in the frequency range *f* ≈ 2 – 2.5 THz. Maximum width of the radiation spectrum in this tuning area occurs for *U* = 57 mV/period, where we have Δ*ν̂*_*max*__#1F_ = 5149 cm^–1^ and this corresponds to changes *f′*_*in#*__1F_ = 2.47 THz to *f′*_*max#*__1F_ = 4.01 THz. Throughout the tuning region of the QCL_TR#1F_, radiation from 2.47 (for *U* = 57 mV/period) to 5.21 THz (for *U* = 78 mV/period) can be obtained.

The most visible effect of widening the quantum well of the injector by one monolayer (*Q*_*w*_ + 0.6 nm) is the narrowing of the radiation spectrum. Interestingly, in a large part of the QCL_TR#2F_ (for *U* = 55 – 75 mV/period), the radiation spectrum of structure #2 does not extend beyond the boundaries of the radiation spectrum of structure #0. The shift of the spectrum toward lower frequencies (relative to Figure 6c), can only be seen for narrow intervals at the beginning and end of the tuning area (for *U* = 35–38 mV/period and *U* = 75–78 mV/period) as well as for supply voltages in the *U* = 45–55 mV/period range. For example, for the supply voltage *U* = 78 mV/period in Figure 6d) one can read the changes in the emitted radiation frequencies in the range *f*_*min#*__2F_ = 3.12 THz to *f*_*max#*__2F_ = 3.41 THz. That is, below the range *f*_*min*__#0F_ = 3.6 THz to *f*_*max*__#0F_ = 3.85 THz, characteristic of the original laser structure. However, the width of the radiation spectrum for the same voltage is Δ*f*_#2F_ = 0.29 THz (in conversion Δ*ν̂*_#2F_ = 968 cm^–1^), which is more than twice the value of Δ*f*_#0F_ = 0.12 THz (in conversion Δ*ν̂*_#0F_ = 403 cm^–1^). The broadest spectrum in the Q_TR#2F_ tuning range is observed at *U* = 76 mV/period, where the device emits radiation in the range of *f*_#2F_ = 2.95 – 4.69 THz, resulting in Δ*f*_*max*__#2F_ = 1.74 THz (converted Δ*ν̂*_*max*__#2F_ = 5807 cm^–1^). This spectrum completely includes the Δ*ν̂*_#0F_ = 2134 cm^–1^ which extends in the frequency range *f*_#0F_ = 3.48 – 4.12 THz for original structure. The entire Q_TR#2F_ tuning region covers radiation from 1.85 THz (for *U* = 35 mV/period) to 4.52 THz (for *U* = 77 mV/period).

A general review of the results obtained with the FMSL (see Table 2) shows that narrowing the injector well by one monolayer did not significantly change the trend of changes in emitted radiation as a function of supply voltage. However, a greater width of the frequency tuning range was obtained, which is represented here by the parameter TR_fw_ (tuning range frequency width) defined as the difference of the highest and lowest frequency in the tuning region (TR_fw_ = 5.21 – 2.47 = 2.74 THz).

**Table 2:** Selected Tuning Parameters of Tested QCL Structures Calculated Using FMSL

	structure #1	structure #0	structure #2
tuning area	QCL_TR#1F_	QCL_TR#0_	QCL_TR#2F_
frequecy range (THz)	2.47–5.21	1.9–4.4	1.85–4.52
TR_fw_ (THz)	2.74	2.5	2.67
wavelengths (μm)	121 to 57.5	158 to 6.8.1	162 to 66.3
tunning voltage range (mV/period)	42–78	38–78	35–78
TR_SI,_ (mV/period)	36	40	43
Δ*ν̂*_*max*_ (cm^–1^)	6000	6800	5807

For comparison, in structure #0, we get TR_fw_ = 4.4 – 1.9 = 2.5 THz. On the other hand, the range of the supply voltage controlling the QCL_TR#1F_ area has been slightly reduced, as indicated by the TR_SI_ (tuning range supply interval) parameter defined as the difference of the largest and smallest value of the supply voltage in the tuning area under consideration. For structure #1 it is TR_SI_ = 78 – 42 = 36 mV/period for comparison in structure #0 we have TR_SI_ = 78 – 38 = 40 mV/period. Similarly, the largest observed spectral width of radiation (Δ*ν̂*_*max*_) in the QCL_TR#1F_ area exhibits a 12% decrease in value when compared to the parameter’s value in the QCL_TR#0_ area.

The situation is somewhat different for the injector well broadening. It turns out that in this case we have a slightly smaller broadening of the tuning region in the frequency range (TR_SI_ = 2.67 THz), but with an increased control voltage interval (TR_SI_ = 43 mV/period) and a slightly smaller maximum width of the radiation spectrum (Δ*ν̂*_*max*_ = 5807 cm^–1^). The results also showed that there were three distinct changes in this trend in the QCL_TR#2F_ region (around *U* = 44, 60, 70 mV/period), so calculations using RSM were necessary to verify it thoroughly. The simulation results in this range are shown in Figure 7c,d, where there are maps of optical gain as a function of supply voltage and frequency of emitted photons calculated for the temperature *T* = 200 K. In addition, selected parameters of the tuning regions of the tested QCL structures calculated with RSM are listed in Table 3.

**Table 3:** Selected Tuning Parameters of Tested QCL Structures Calculated Using RSM

	structure #1	structure #0	structure #2		
tuning area	QCL_TR#1R_	QCL_TR#0_	QCL_TR#2a_	QCL_TR#2b_	QCL_TR#2c_
frequecy range (THz)	2.47–4.41	1.9–4.4	2.31–3.74	3.42–4.24	2.0–2.4
TR_fw_ (THz)	1.94	2.5	1.43	0.82	0.4
wavelengths (μm)	121 to 68.0	158 to 68.1	130 to 80.2	87.7 to 70.7	150 to 125
tunning voltage range (mV/period)	51–72	38–78	43–60	62–72	58–64
TR_SI_ (mV/period)	21	40	17	10	6
Δ*ν̂*_*max*_ (cm^–1^)	5337	6800	4170	1835	1334

For the narrowed injector well, the trend line of changes in *hν*_*mG*_ (see Figure 7c) does not differ significantly from what was observed for the original structure (see Figure 5c). Assuming *g*_sb_ ≅ 40 cm^–1^, the tuning region QCL_TR#1R_ can be determined, which covers the frequency range *f*_TR#1R_ = 2.47 – 4.41 THz (for *U* = 51–72 mV/period), which corresponds to waves with lengths λ_TR#1R_ = 68–121 μm. The maximum width of the radiation spectrum occurs here for *U* = 60 mV/period and is Δ*ν̂*_*max*__#1R_ = 5337 cm^–1^, which, in relation to the previously given value calculated using FMSL, informs us about a slight shift of the maximum spectrum width toward a higher supply voltage. At the same time, by comparing the QCL_TR#0R_ and QCL_TR#1R_ areas, it can be concluded that, as predicted by FMSL, there was a shift in the laser tuning range toward higher frequencies. Finally, after careful calculations taking into account all major electron scatters, it is found that narrowing the injector area reduces the frequency tuning range (TR_fw_ = 1.94 THz) and the control voltage interval (TR_SI_ = 21 mV/period) with respect to the original #0 structure.

For the widened injector well, the *hν*_*mG*_ trend line shows significant changes compared to that observed for the original structure, as can be seen in Figure 7d. This is because the optical gain map shows three separate regions for which *g* > 40 cm^–1^ (QCL_TR#2*a*_, QCL_TR#2*b*_ and QCL_TR#2*c*_). They refer to changes in the trend of the dependence of the emission frequency on the supply voltage QCL, predicted using FMSL (see Figure 7a) and they occur for similar voltages as in the mentioned case, so we have peaks of optical gain for *U* = 44, 60, 77 mV/period. This makes the laser tuning for this case more complex and covers three ranges with different spectra and light intensity.

The largest of the above ranges is QCL_TR#2*a*_, within which the laser can be tuned in the range *f*_TR#2*a*_ = 2.3 – 3.74 THz (for *U* = 43–60 mV/period), corresponding to wavelengths λ_TR#2*a*_ = 80.2–130.3 μm. This gives TR_fw_ = 1.43 THz for the control voltage range TR_SI_ = 17 mV/period. The maximum width of the radiation spectrum is Δ*ν̂*_*max*__#2a_ = 4170^–1^ for *U* = 48 mV/period (FMSL predicts Δ*ν̂*_#2F_ = 4274 cm^–1^ for *U* = 44 mV/period). The tuning range of QCL_TR#2*b*_ covers the frequencies *f*_TR#2*b*_ = 3.42 – 4.24 THz (for *U* = 62 – 72 mV/period), which corresponds to the wavelengths λ_TR#2*b*_ = 70.7 – 87.7 μm. This results in a TR_fw_ = 0.82 THz for the control voltage range of TR_SI_ = 10 mV/period. The maximum width of the radiation spectrum occurs here for *U* = 66 mV/period and is Δ*ν̂*_*max*__#2*b*_ = 1835 cm^–1^. The trend change predicted by FMSL occurs for *U* = 78 mV/period at Δ*ν̂*_#2F_ = 1613 cm^–1^, but is as high as Δ*ν̂*_#2F_^’’^ = 5081 cm^–1^ for *U* = 76 mV/period. Such a significant difference in spectral width observed in the FMSL results can be attributed to the presence of an additional tuning range (QCL_TR#2*c*_). Assuming *g*_*sb*_ ≅ 40 cm^–1^, the frequency of *f*_TR#2*c*_ changes from 2 to 2.4 THz (TR_fw_ = 0.4 THz), corresponding to wavelengths λ_TR#2*c*_ = 124.9–149,9 μm at a supply voltage *U* = 58–64 mV/period (TR_SI_ is only 6 mV/period). As can be seen, the range of supply voltages associated with tuning in the QCL_TR#2c_ area partially covers the ranges of control voltages of the QCL_TR#2*a*_ and QCL_TR#2*b*_ areas, which limits the possibility of seeing this phenomenon only in optical gain energy maps. The maximum spectral width of the radiation of the QCL_T#2*c*_ area occurs at *U* = 61 mV/period and is equal to Δ*ν̂*_*max*__#2*c*_ = 1334 cm^–1^ (predicted by FMSL for *U* = 60 mV/period, where Δ*ν̂*_#2F_^’’’^ = 1631 cm^–1^).

## 5 Experiment Hint

**Figure 8:**
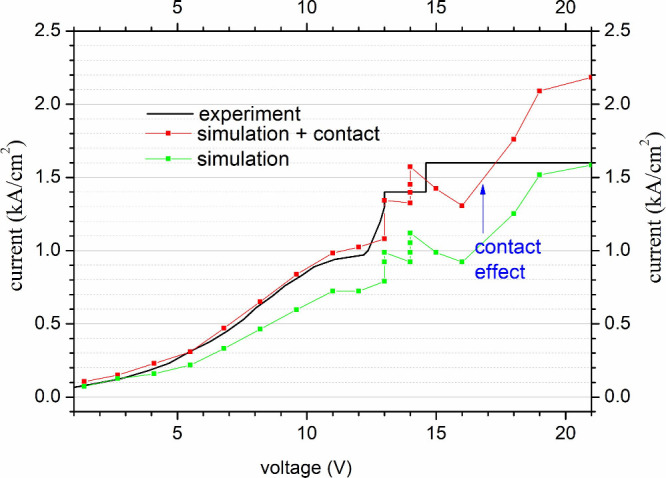
Experimental *I*/*V* characteristics (black solid line) measured and described in ref (59) related to the calculated characteristics (red line + symbol), which was created after shifting the *I*/*V* simulation (green line + symbol graph) taking into account the series resistance of 11 Ω.

In order to verify the simulation of the tested QCL device against its measurements, a comparison was made between the measured and simulated current–voltage characteristics. The results are shown in Figure 8, where the black solid line shows the characteristics obtained by the experiment described in the paper,^59^ and the curve of the corresponding I/U relationship (green line + symbol plot) calculated using the RSM model. As can be seen, the calculated current–voltage characteristic differs from the experimental one. This fact is well-known and described in the literature ^66^ and ^67^ and results from the existence of parasitic effects such as series resistance or voltage drop at the metal–semiconductor interface.

After taking these effects into account (red line + symbol graph), a good agreement between simulations and measurements was obtained. However, it should be emphasized that such a situation is only possible in the subthreshold range, since the RSM model based on NEGF does not take into account light-matter interactions.

Simulation results for the original structure #0 also showed that the maximum gain for the temperature *T* = 200 K corresponds to a frequency in the range of 3.4–3.6 THz and agrees with the measured frequency of ∼3.6 THz, and the value of this gain is close to the standard mirror and waveguide losses in this type of laser. This means that the temperature *T* = 200 K is the emission limit temperature of this laser as measured by^59^ (*T*_*max*_ = 199.5 K).

## 6 Conclusions

The conducted tests confirmed the apparent sensitivity of the QCL radiation designed by Kumar et al.^60^ to changing the width of the injector well. Depending on the thickness of the injector layer, it is possible to obtain either homogeneous (QCLTR#0, QCLTR#1) or nonhomogeneous (QCLTR#2) tuning regions with a linear or nonlinear trend of *hν*_*mG*_ changes. Despite apparent changes in the width of the radiation spectrum due to changes in the width of the injector well, no large changes were observed in the frequency range of the emitted wavelengths. It is a well-known feature of terahertz lasers that it is difficult to achieve a significant shift in the radiation spectrum without significant changes in the superlattice structure. However, two examples of the device (structures #1 and #2) were proposed, which with a small change in the width of the injector layer (by one monolayer) can have different tuning characteristics. A homogeneous one with a wide radiation spectrum can be used in systems for detecting and imaging a wide variety of objects, while a nonhomogeneous one with several smaller radiation spectral widths can be used for more precise imaging of selected targets, but this case requires a complex laser power control system. The work performed showed the important role of the developed numerical models (FMSL and RSM), which allowed effective and accurate simulations of demanding QCL structures. In summary, the main achievement of our work is the proposal of a precise modeling of the influence of the injector layer on the emission wavelength control, which has not been analyzed in such detail in the literature.

## Abbreviations

QCL: quantum cascade laser
FMSL: finite model of superlattice
MBM: modified bisection method
NDR: negative dynamic resistance
RSM: real space model
THz-TDS: terahertz time-domain spectroscopy
WFM: Wannier function method
